# Age- and sex-specific prevalence and ten-year risk for cardiovascular disease of all 16 risk factor combinations of the metabolic syndrome - A cross-sectional study

**DOI:** 10.1186/1475-2840-9-34

**Published:** 2010-08-09

**Authors:** Susanne Moebus, Chakrapani Balijepalli, Christian Lösch, Laura Göres, Bernd von Stritzky, Peter Bramlage, Jürgen Wasem, Karl-Heinz Jöckel

**Affiliations:** 1 Institute for Medical Informatics, Biometry and Epidemiology, University Hospital of Essen, University Duisburg-Essen, Germany; 2 Sanofi-Aventis Deutschland GmbH, Berlin, Germany; 3 Institute for Cardiovascular Pharmacology and Epidemiology, Mahlow, Germany; 4 Alfried Krupp von Bohlen und Halbach Foundation, Institute for Healthcare Management, Faculty of Economics, University of Duisburg-Essen, Germany

## Abstract

**Background:**

Based on the AHA/NHLBI-definition three out of five cardiometabolic traits must be present for the diagnosis of the metabolic syndrome (MetS), resulting in 16 different combination types. The associated cardiovascular risk may however be different and specific combination may be indicative of an increased risk, furthermore little is known to which extent these 16 combinations contribute to the overall prevalence of MetS. Here we assessed the prevalence of all 16 combination types of MetS, analyzed the impact of age and gender on prevalence rates, and estimated the 10-year risk of fatal and non-fatal myocardial infarction (MI) of each MetS combination type.

**Methods:**

We used data of the German Metabolic and Cardiovascular Risk Project (GEMCAS), a cross-sectional study, performed during October 2005, including 35,869 participants (aged 18-99 years, 61% women). Age-standardized prevalence and 10-year PROCAM and ESC risk scores for MI were calculated.

**Results:**

In both men and women the combination with elevated waist-circumference, blood pressure and glucose (WC-BP-GL) was the most frequent combination (28%), however a distinct unequal distribution was observed regarding age and sex. Any combination with GL was common in the elderly, whereas any combination with dyslipidemia and without GL was frequent in the younger. Men without MetS had an estimated mean 10-year risk of 4.7% (95%-CI: 4.5%-4.8%) for MI (PROCAM), whereas the mean 10-year risk of men with MetS was clearly higher (age-standardized 7.9%; 7.8-8.0%). In women without MetS the mean 10-year risk for MI was 1.1%, in those with MetS 2.3%. The highest impact on an estimated 10-year risk for MI (PROCAM) was observed with TG-HDL-GL-BP in both sexes (men 14.7%, women 3.9%). However, we could identify combinations with equal risks of non-fatal and fatal MI compared to participants without MetS.

**Conclusions:**

We observed large variations in the prevalence of all 16 combination types and their association to cardiovascular risk. The importance of different combinations of MetS changes with age and between genders putting emphasis on a tailored approach towards very young or very old subjects. This knowledge may guide clinicians to effectively screen individuals and prioritize diagnostic procedures depending on age and gender.

## Background

The metabolic syndrome (MetS) is defined as a cluster of risk factors which predispose an individual to diabetes mellitus type 2 and cardiovascular disease [1,2]. According to the most widely used definition for the diagnosis of MetS - the National Cholesterol Education Program Adult Treatment Panel (NCEP ATP) III [3] and their modification from the AHA/NHLBI [4] - at least three out of five cardiovascular or cardiometabolic traits must be present to diagnose MetS: (1) elevated blood glucose or known diabetes mellitus (GL), (2) increased waist circumference (WC), (3) increased triglycerides (TG), (4) low high density lipoprotein cholesterol (HDL) and (5) elevated blood pressure (BP). This definition allows 16 possible combinations for a diagnosis of MetS.

There is a controversial debate about the usefulness of the concept of the MetS [5-10]. One of the criticisms refers to underlying implicit assumption that each combination type of the MetS uniformly increases risk for cardiovascular outcomes. Numerous studies addressed partly this issue by investigating the effect of the number of MetS-defining traits. These studies show a gradual increase in risk of cardiovascular outcomes with numerically increasing traits [11,12]. Accordingly, previous studies reported a progressive increase for cardiovascular outcomes in those individuals diagnosed with MetS consisting of 5 traits compared to those diagnosed with MetS consisting of only 3 traits [13-17].

However, epidemiologic data on the distribution of the 16 combination types are sparse and little is known to which extent these 16 combinations contribute to the overall prevalence of MetS. Previous studies have focused on the prevalence of the number of MetS components. To the best of our knowledge there has been only one study that compared the prevalence of all MetS combinations. This study included 4 different studies from the Asia-Pacific region [18]. Furthermore, prevalence data according to age, sex and cardiovascular disease are missing and not much is known, whether different combinations translate into a differential risk for the incidence of cardiovascular events [19]. Usually such detailed analyses are hampered by small sample sizes. The knowledge of these associations might mandate physicians to effectively screen subjects and prioritize diagnostic steps depending on age and gender.

The German Metabolic and Cardiovascular Risk Project (GEMCAS) is a point prevalence study including 35,869 participants with an age-range of 18-99 years, aiming to determine the prevalence of the MetS in Germany [20,21]. The present analysis was undertaken (1) to determine the prevalence of the 16 possible combination types of MetS according the AHA/NHLBI definition, (2) to analyze the impact of age and gender on prevalence, and (3) to associate the different combination types to the 10-year risk of fatal and non-fatal cardiovascular disease based on PROCAM and ESC score estimates.

## Methods

### Design and subjects

GEMCAS was conducted during two weeks in October 2005 at 1,511 randomly selected primary care physicians across Germany. Physicians specialized in cardiology and/or diabetes treatments were excluded because of the assumed higher prevalence of the MetS in their clientele. Methods have been previously described in detail [20,21]. In short, all eligible subjects aged ≥18 years visiting a general practitioner regardless of the reason of their visit were included. The study was planned and conducted according to the German guidelines for Good Epidemiology Practices (GEP) [22]. All participants gave their written informed consent and the study protocol was approved by the institutional ethics committee.

### Data collection

Data were collected on sociodemographic variables, smoking habits, and lifestyle aspects. The participating physicians recorded a history of diabetes mellitus (DM) and cardiovascular disease (CVD), the latter defined as myocardial infarction and stroke. Further a standardized assessment according to the study protocol for all risk factors required for diagnosis of MetS - WC, BP, GL, TG and HDL - was carried out. For blood sampling a two-step approach was performed [21]. First, all subjects were included regardless of fasting status. By using a blood glucose quick test, it was possible to directly exclude or diagnose hyperglycaemia, independent of the subject's fasting state, based on the selected capillary blood glucose concentration cut points of <5.56 or >11.11 mmol/L. If the findings concerning blood glucose or triglycerides were ambiguous due to a meal in the previous 8 respective 12 hours, the subject was asked to come for a second appointment to give a fasting blood sample. All blood samples were shipped within 24 hours to a central laboratory (Berlin, Germany) by an assigned courier service. Participating physicians were equipped from the central laboratory with tubes containing the glycolysis inhibitor sodium fluoride, which ensured that the samples could be stored at room temperature for up to 24 hours [23].

### Quality assurance

To reduce the logistics of coordinating such a large, country-wide study, participating GPs received no other instructions than the information material sent to them by mail prior to the survey. To control for a proper procedure and to ensure the robustness of the data obtained, a specifically adapted monitoring system was designed to meet the high logistic needs [21]. This monitoring concept included telephone-monitoring and random on-site visits. Telephone monitoring was performed at >50% of the enrolled sites prior to the day of the survey to ensure all participating physicians had the complete and correct set of forms, documents and blood sampling materials available on the survey day. Physicians to be included in telephone monitoring were selected randomly but stratified by the 3 recruitment groups. After the phone call the interviewers rated the monitored site based on the interview results. On-site visits were performed on the day of the survey, with a focus on quality of data and measurements. Monitoring visits were planned in at least 10% of the participating medical practices. A priori, these practices were chosen at random though proportional to the 3 recruitment groups. Some sites that received a poor rating after the telephone monitoring were to be specifically included in the on-site monitoring, if possible.

### Definition of the MetS

We defined MetS according to AHA/NHBLI 2004 [4]. This includes the presence of any three of the following five traits: (a) WC > 102 cm in men or >88 cm in women, (b) TG ≥ 1.7 mmol/L (150 mg/dl), (c) HDL < 1.08 mmol/L (40 mg/dl) in men and <1.3 mmol/L (50 mg/dl) in women, (d) BP ≥ 130/≥85 mmHg and (e) fasting GL ≥ 5.6 mmol/L (100 mg/dl) or random GL ≥ 11.1 mmol/L (200 mg/dl) or known diabetes. In accordance with the definition of 2004, we have performed the following adjustments: We used here the recommended lowered cutpoint of GL ≥ 5.6 mmol/L (100 mg/dl) according to a footnote in table 1 of the original publication [4]). Furthermore we included a history of diabetes and a random GL > 11.1 mmol/L since in the NHLBI/AHA Conference Proceedings 2004 it was claimed, that the primary clinical outcome MetS was identified as CVD [4].

### Statistical analyses

For all analyses SAS (Version 9.2) was used. Subjects' characteristics were calculated as means for continuous variables and frequency for all categorical variables. Age-standardized prevalence rates were also calculated to compare the prevalence of cardiovascular comorbidities with the different combinations. We used direct age-standardization and their 95%-confidence intervals (CI) [24] according to the German population 2004 [25]. To avoid minimal cell counts we categorized age in following groups: 18-40, 41-60, 61-80, and 81-99.

Finally we analyzed the association of all 16 combinations with the 10-year risk of fatal and nonfatal myocardial infarction (MI) using the PROCAM score for men (aged 35-65 years) and modified for women (aged 45-65 years) [26]. The ESC Score for men (aged 35-65 years) and for women (aged 45-65 years) was calculated as well [27]. We calculated age-standardized risk estimates stratified by sex, using the direct standardization with age groups (according to PROCAM and ESC) 35-42, 43-49, 50-57 and 58-65 in men; and 45-51, 52-58 and 59-65 in women, respectively. We used participants without MetS as the standard population.

For all combination analysis, we performed complete case analyses and made sure that subjects falling under one combination of the higher order (e.g. 4 risk factors present) are not simultaneously falling under the combinations with lower order and less criteria (e.g. any 3 of the above mentioned 4).

## Results

### Baseline characteristics

GEMCAS included 13,942 men (38.9%) and 21,927 women with a mean age of 53 and 50.9 years respectively (Table 1). In 2,768 participants MetS was not definable because of missing traits. About 70% of men and 54% of women either were overweight (25-30 kg/m^2^) or obese (≥30 kg/m^2^). Current smokers were 27.5% of men and 23.5% of women and 20.5% of men and 10.5% of women had a history of cardiovascular disease.

The mean age of subjects with MetS was higher for both genders as was the degree of obesity (Table 1). Levels of TG, GL and BP and average intake of medication (drugs against hypertension and dyslipidemia) were generally higher in subjects with MetS compared to those without MetS.

**Table 1:** Characteristics of the total study population and individuals with and without the Metabolic Syndrome

Risk factor/Trait	All (n = 35,869)		Without MetS* (n = 25,961)		With MetS* (n = 7,122)	
	**Men**	**Women**	**Men**	**Women**	**Men**	**Women**

N (%)	13,942 (38.9)	21,927 (61.1)	9,452 (36.4)	16,509 (63.6)	3,168 (44.5)	3,954 (55.5)
Age (years), mean ± SD	53.0 ± 15.8	50.9 ± 16.2	50.9 ± 16.3	48.1 ± 15.9	58.8 ± 12.9	60.5 ± 13.9
Weight (kg), mean (±SD)	86.2 ± 14.9	71.9 ± 15.2	81.7 ± 12.2	68.3 ± 13.1	97.1 ± 15.3	84.5 ± 15.9
Body Mass Index (%)						
≤25 kg/m^2^	29.7	46.4	40.4	57.9	4.3	8.0
25 - <30 kg/m^2^	45.6	30.3	47.7	28.9	38.6	33.1
≥30 kg/m^2^	24.7	23.3	11.9	13.1	57.1	58.9
Waist circumference (cm), mean ± SD	98.8 ± 13.0	86.8 ± 14.3	94.1 ± 10.9	82.5 ± 12.0	110.4 ± 10.7	101.6 ± 11.8
Blood parameters						
Blood glucose (mmol/l), mean ± SD	5.7 ± 2.0	5.3 ± 1.6	5.2 ± 1.4	4.9 ± 0.9	7.1 ± 2.9	6.7 ± 2.6
Total Cholesterol (mmol/l), mean ± SD	5.3 ± 1.1	5.4 ± 1.1	5.2 ± 1.0	5.3 ± 1.0	5.3 ± 1.2	5.6 ± 1.1
HDL (mmol/l), mean ± SD	1.4 ± 0.4	1.7 ± 0.4	1.5 ± 0.4	1.8 ± 0.4	1.2 ± 0.3	1.4 ± 0.4
LDL (mmol/l), mean ± SD	3.3 ± 0.9	3.3 ± 0.9	3.3 ± 0.9	3.2 ± 0.9	3.3 ± 1.0	3.5 ± 1.0
Triglycerides (mmol/l), median(Q1; Q3)	1.6 (1.1;2.4)	1.3 (0.9;1.9)	1.4 (1.0;1.9)	1.2 (0.9;1.5)	2.4 (1.7;3.4)	2.1 (1.6;2.9)
Blood Pressure (BP)						
Systolic BP (mmHg), mean ± SD	133.6 ± 18.2	128.5 ± 19.3	129.9 ± 17.4	124.4 ± 17.9	142.7 ± 16.8	142.7 ± 17.5
Diastolic BP (mmHg), mean ± SD	81.4 ± 10.4	79.2 ± 10.6	79.9 ± 10.0	77.6 ± 10.2	84.9 ± 10.5	84.5 ± 10.4
Smoking status (%)						
Current Smoker	27.5	23.5	28.7	24.6	23.7	18.9
Past Smoker	39.8	22.3	35.4	22.4	50.3	21.6
Never Smoker	32.8	54.2	35.9	53.0	26.0	59.5
MetS Traits (%)						
WC	36.4	41.5	17.1	26.3	84.1	92.8
TG	24.0	12.9	12.1	4.6	63.7	53.4
HDL	12.2	13.9	4.6	5.6	32.8	46.8
BP	66.6	52.9	55.8	41.8	92.5	91.6
GL	29.5	17.6	13.1	5.3	75.6	68.1
Pharmacotherapy (%)						
Anti-diabetic	12.0	7.0	4.9	1.6	34.2	30.1
Anti-hypertensive	41.7	34.2	32.3	25.0	67.3	67.8
Lipid lowering	18.1	10.5	14.0	6.9	29.5	24.6
Comorbidities (%)						
Cardiovascular diseases	20.5	10.5	16.5	7.2	31.7	22.9
Cancer	5.5	4.9	5.2	4.3	6.7	7.1

*Of n = 2,768 subjects MetS was not definable because of missing traits

MetS, Metabolic Syndrome (for cut points used see methods); BMI, Body Mass Index; SD, Standard Deviation; HDL, High Density Lipoprotein; LDL, Low Density Lipoprotein; BP, Blood Pressure; WC, waist circumference; TG, triglyceride; BP, blood pressure; GL, blood glucose

### Frequency of risk factor combinations

**Figure 1:**
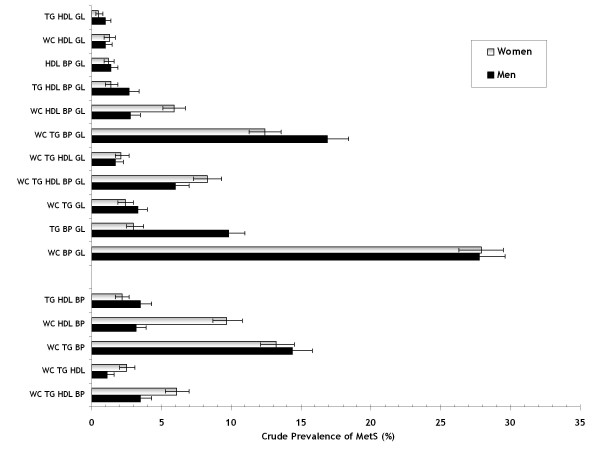
**Prevalence (crude) of all 16 combinations in subjects with MetS**. In the lower columns: 5 combinations more prevalent in the young, upper columns: 11 combinations more prevalent in the elderly.

Three traits had 66% of men and 64% of women, 27/28% had four traits and 6%/8% had 5 traits. Figure 1 displays the distribution of the different combinations that make up the MetS and their relative frequency. WC-BP-GL was the most frequent combination in both men and women (both 28%). The combination of WC-HDL-BP was much more frequent in women than in men (10 vs. 3%), whereas TG-BP-GL was much more frequent in men than women (10 vs. 3%). In women, the 8 combinations with the highest prevalence involved an elevated blood pressure (in men 7 out of 8 combinations). In both sexes, 5 out of 8 involved an increased waist circumference.

In Table 2 the prevalence of combinations is presented by sex and age-groups. The combination of WC-TG-BP was most frequent in younger men (18-30 years: 38%; 31-46: 22%; 46-60: 17%; 61-75: 10%; >76 years: 8%), almost the same combination was most frequent in older men, except that TG was exchanged by GL (WC-BP-GL: 2, 15, 26, 34, 36% resp.). Interestingly, none of the combinations types most frequent in the younger men had an elevated blood glucose included, all combinations types including elevated blood glucose were more prevalent in the elderly - with the exception of those combinations types not including elevated blood pressure. The prevalence of these combinations was either not different between younger and older men or tended to be higher in younger men.

**Table 2:** Prevalence of all 16 combination types according to age-groups and sex

Combinations of MetS					18-30 years **% (95%-CI)**^**1**^		31-45 years % (95%-CI)		46-60 years % (95%-CI)		61-75 years % (95%-CI)		76-99 years % (95%-CI)	
**WC**	**TG**	**HDL**	**BP**	**GL**										

**Combinations more frequent in the young**					**Men**	**Women**	**Men**	**Women**	**Men**	**Women**	**Men**	**Women**	**Men**	**Women**

**WC**	**TG**	**HDL**	**BP**	**-**	10.9 (4.5-21.3)	**13.3** (6.8-22.5)	9.0 (6.1-12.7)	**10.5** (7.6-14.0)	3.3 (2.2-4.6)	**7.4** (5.9-9.2)	1.9 (1.1-2.9)	**4.1** (3.1-5.4)	1.8 (0.5-4.4)	**3.5** (2.0-5.6)
**WC**	**TG**	**HDL**	**-**	**-**	0	**9.6** (4.3-18.1)	3.7 (1.9-6.4)	**7.6** (5.2-10.8)	1.5 (0.8-2.5)	**2.7** (1.8-3.9)	0.3 (0.1-0.9)	**1.0** (0.5-1.7)	0	**0.7** (0.1-1.9)
**WC**	**TG**	**-**	**BP**	**-**	**37.5** (25.7-50.5)	13.3 (6.8-22.5)	**22.4** (17.9-27.3)	12.6 (9.4-16.4)	**16.5** (14.2-19.0)	14.7 (12.6-17.0)	10.0 (8.2-12.0)	**13.0** **(11.2-15.0)**	7.5 (4.4-11.7)	**11.1** (8.4-14.4)
**WC**	**-**	**HDL**	**BP**	**-**	12.5 (5.6-23.2)	**39.8** (29.2-51.1)	6.5 (4.1-9.8)	**27.6** (23.1-32.3)	2.0 (1.2-3.1)	**9.7** (8.0-11.7)	2.5 (1.6-3.7)	**4.3** (3.3-5.6)	3.5 (1.5-6.8)	**3.7** (2.2-5.9)
**-**	**TG**	**HDL**	**BP**	**-**	**18.8** (10.1-30.5)	2.4 (0.3-8.4)	**10.3** (7.2-14.1)	5.2 (3.2-8.0)	**2.9** (1.9-4.1)	1.9 (1.1-2.9)	1.3 (0.7-2.2)	**1.9** (1.2-2.8)	**2.2** (0.7-5.0)	1.1 (0.4-2.5)
Combinations more frequent in the elderly														
**WC**	**-**	**-**	**BP**	**GL**	1.6 (0.0-8.4)	**4.8** (1.3-11.9)	**14.6** (10.9-18.9)	10.8 (7.8-14.3)	25.9 (23.1-28.8)	**27.0** (24.3-29.8)	**33.8** **(30.9-36.9)**	33.7 (31.1-36.5)	**35.5** (29.3-42.1)	32.7 (28.4-37.2)
**-**	**TG**	**-**	**BP**	**GL**	0 -	0 -	**8.7** (5.9-12.3)	0.8 (0.2-2.3)	**10.3** (8.5-12.5)	2.6 (1.7-3.8)	**10.2** (8.4-12.3)	2.7 (1.9-3.8)	**10.1** (6.5-14.8)	7.2 (5.0-10.0)
**WC**	**TG**	**-**	**-**	**GL**	0 -	1.2 (0.0-6.5)	**1.6** (0.5-3.6)	1.6 (0.6-3.4)	**4.4** (3.2-6.0)	2.0 (1.3-3.1)	3.1 (2.1-4.4)	**3.1** **(2.2-4.2)**	**2.6** (1.0-5.6)	2.2 (1.1-4.0)
**WC**	**TG**	**HDL**	**BP**	**GL**	3.1 (0.4-10.8)	**6.0** (2.0-13.5)	3.7 (1.9-6.4)	**5.2** (3.2-8.0)	7.1 (5.5-8.9)	**8.4** (6.8-10.2)	6.3 (4.9-8.0)	**8.7** (7.2-10.5)	4.8 (2.4-8.5)	**9.6** (7.1-12.7)
**WC**	**TG**	**HDL**	**-**	**GL**	0 -	0 -	2.2 (0.9-4.4)	**3.4** (1.8-5.8)	1.3 (0.7-2.2)	**2.6** (1.7-3.8)	**2.1** (1.3-3.2)	1.8 (1.1-2.7)	1.3 (0.3-3.8)	**1.3** (0.5-2.8)
**WC**	**TG**	**-**	**BP**	**GL**	**4.7** (1.0-13.1)	1.2 (0.0-6.5)	**9.6** (6.6-13.4)	3.9 (2.2-6.4)	**16.4** (14.1-18.9)	12.3 (10.3-14.4)	**20.0** (17.6-22.6)	15.7 (13.7-17.9)	**18.9** (14.0-24.6)	12.9 (9.9-16.3)
**WC**	**-**	**HDL**	**BP**	**GL**	1.6 (0.0-8.4)	**6.0** (2.0-13.5)	1.2 (0.3-3.2)	**6.3** (4.1-9.2)	2.3 (1.5-3.5)	**5.3** (4.0-6.8)	3.8 (2.7-5.2)	**6.0** (4.8-7.5)	2.6 (1.0-5.6)	**6.3** (4.3-9.0)
**-**	**TG**	**HDL**	**BP**	**GL**	**1.6** (0.0-8.4)	0 -	**3.4** (1.7-6.0)	0.5 (0.1-1.9)	**2.6** (1.7-3.9)	0.7 (0.3-1.4)	**2.4** (1.5-3.5)	1.6 (1.0-2.5)	3.1 (1.2-6.2)	**3.3** (1.8-5.3)
**-**	**-**	**HDL**	**BP**	**GL**	**3.1** (0.4-10.8)	1.2 (0.0-6.5)	0.6 (0.1-2.2)	**1.0** (0.3-2.7)	**1.4** (0.7-2.3)	0.7 (0.3-1.4)	**0.9** (0.4-1.7)	0.8 (0.4-1.5)	**4.4** (2.1-7.9)	3.5 (2.0-5.6)
**WC**	**-**	**HDL**	**-**	**GL**	**1.6** (0.0-8.4)	1.2 (0.0-6.5)	1.6 (0.5-3.6)	**1.8** (0.7-3.8)	1.1 (0.5-1.9)	**1.4** (0.8-2.3)	0.9 (0.4-1.7)	**1.2** (0.7-2.0)	0.4 (0.0-2.4)	**0.7** (0.1-1.9)
**-**	**TG**	**HDL**	**-**	**GL**	**3.1** (0.4-10.8)	0 -	0.9 (0.2-2.7)	**1.0** (0.3-2.7)	**1.2** (0.6-2.1)	0.7 (0.3-1.4)	**0.6** (0.2-1.3)	0.2 (0.1-0.7)	**1.3** (0.3-3.8)	0.4 (0.1-1.6)

WC, waist circumference; TG- triglycerides; HDL, High Density Lipoproteins; elevated BP, blood pressure; GL, elevated blood glucose. For cut points used see methods; 95%-CI, 95% confidence intervals; ***the higher prevalence in comparing men and women is in bold;***

^**1 **^Percent of MetS prevalence in specific group defined by age and gender

In women, a similar observation applied except that WC-HDL-BP was the most frequent combination in younger women, with a large difference between younger and older women (40, 28, 10, 4, 4%, resp.).

A particularly steep incline was observed with age for WC-BP-GL (both genders) with slightly lesser inclines for TG-BP-GL and WC-TG-GL (men > women); these combinations included no subjects with low HDL.

### Combination types and 10-year risk for myocardial infarction (PROCAM)

**Figure 2:**
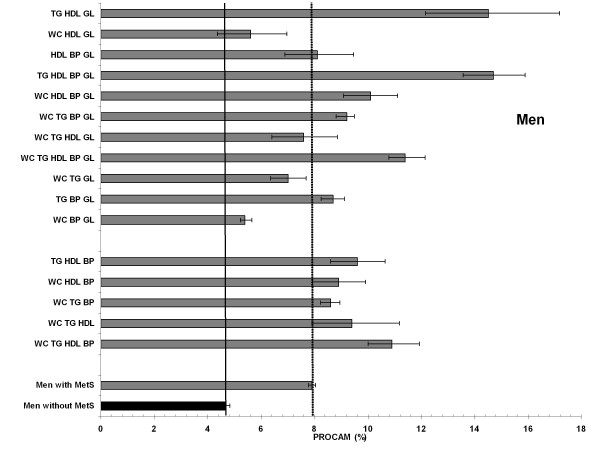
**Age-standardized 10-year risk of myocardial infarction (PROCAM) for men with and men without MetS (reference) and all 16 combinations**. In the lower columns: 5 combinations more prevalent in the young; upper columns: 11 combinations more prevalent in the elderly.

**Figure 3:**
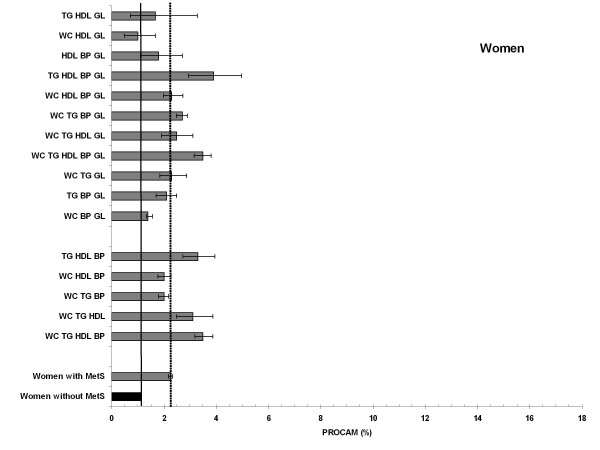
**Age-standardized 10-year risk of myocardial infarction (PROCAM) for women with and without MetS (reference) and all 16 combinations**. In the lower columns: 5 combinations more prevalent in the young; upper columns: 11 combinations more prevalent in the elderly.

According to the PROCAM algorithm the analysis of the 10-year risk for MI for different combination types was computed for the age groups of 35-65 years in men (Figure 2) and 45-65 years in women (Figure 3). Overall, the estimated risk was substantially higher in men than in women throughout every possible combination.

Men without MetS had a mean 10-year risk of 4.7% (95%-CI: 4.5%-4.8%), whereas the mean 10-year risk of men with MetS was clearly higher (age-standardized 7.9%; 7.8%-8.0%). As expected all 16 combinations performed higher risk scores compared to men without MetS (Figure 2). However, we observed large differences between the combinations. Compared to the mean 10-year risk of all men with MetS, three combinations performed significant lower 10-years risk estimates, all including elevated waist-circumference and blood glucose and none of them including elevated blood pressure (WC-BP-GL, WC-TG-GL, and WC-HDL-GL). Furthermore, all three combinations were more frequent in the older men (cf. table 2). The highest 10-year risk for MI in men was found with the combinations TG-HDL-BP-GL (14.7%; 13.6%-15.9%) and TG-HDL-GL (14.5%; 12.2%-17.2%), both not including elevated waist circumference. Interestingly, all combinations that were frequent in the younger men (cf. table 2) resulted in medium to high risk scores (8.6%-11%). Out of the 5 combinations with the highest risk (>10%), 4 included GL, 4 BP and 2 WC.

Women without MetS had a mean 10-year risk of 1.1% (1.1-1.2%), whereas the mean 10-year risk of the women with MetS was twice as high (age-standardized 2.3%). Interestingly, we found two combinations with similar 10-year risks compared to women without MetS (WC-HDL-GL: 1.0%; 0.5%-1.7% and WC-BP-GL: 1.7%; 0.7%-3.3%). As in men, we observed a large variation between the combinations. Compared to the mean 10-year risk of all women with MetS, we observed three combinations with lower 10-years risk estimates (Figure 3), all including elevated waist circumference (WC-HDL-GL, WC-BP-GL, WC-TG-BP), but - different to men - we found one combination with elevated blood pressure. Comparable 10-years risk estimates were observed mainly in older women (WC-TG-GL, TG-BP-GL, WC-HDL-BP-GL and WC-TG-HDL-GL). Noticeably, two of these combinations included four risk factors. The highest estimated risks were observed with the combination TG-HDL-BP-GL (3.9%), WC-TG-HDL-BP and the combination including all five traits (both 3.5%).

In men the estimated 10-year risk for MI for the combination TG-HDL-GL was 9-fold higher compared to women (15.0 vs. 1.7%), and for the combination HDL-BP-GL almost 5-fold higher compared to women (men 8.1%, women 1.8%)

### Combination types and 10-year risk for cardiovascular death (ESC Score)

Since the ESC score predicts fatal cardiovascular events, the ESC risk estimates are much lower compared to the PROCAM risk estimates, latter which predicts fatal and non-fatal events.

**Figure 4:**
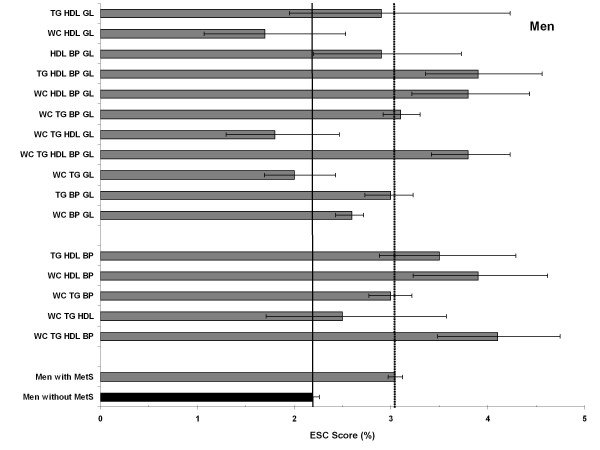
**Age-standardized estimated 10-year risk of fatal cardiovascular disease (ESC) for men with and without MetS (reference) and for all 16 combinations**. In the lower columns: 5 combinations more prevalent in the young; upper columns: 11 combinations more prevalent in the elderly.

Men aged between 35 and 65 years without MetS had an estimated mean 10-year risk of 2.2% (95%-CI: 2.1%-2.3%) for fatal MI, whereas the mean 10-year risk of men with MetS resulted in a mean age-standardized risk of 3.0% (95%-CI: 3.0%-3.1%) (Figure 4). Strikingly, we observed three combinations that resulted in lower risk scores (1.7%-2.0%), compared to the mean risk score of all men without MetS. One of these combinations included even four traits (WC-TG-HDL-GL). All combinations with lower risk estimates include - as in PROCAM - elevated waist-circumference and blood glucose and are more frequent in the older men. However, of those five combinations with the highest scores (>3.8%), four were combinations with 4-5 risk factors, including one risk factor combination which is more prevalent in the younger (WC-TG-HDL-BP, cf. table 2).

**Figure 5:**
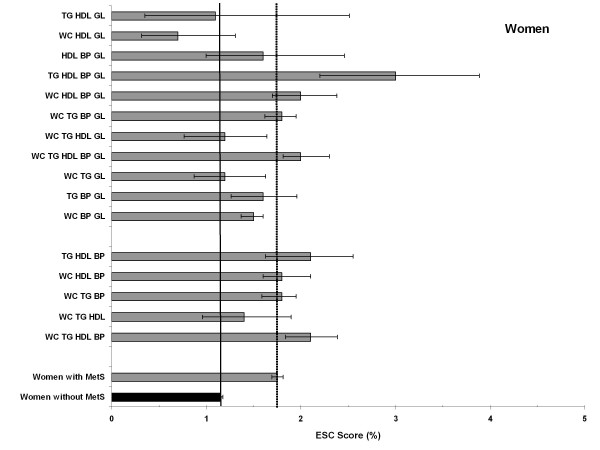
**Age-standardized estimated 10-year risk of fatal cardiovascular disease (ESC) for women with and without MetS (reference) and for all 16 combinations**. In the lower columns: 5 combinations more prevalent in the young; upper columns: 11 combinations more prevalent in the elderly.

We observed a very similar pattern in women, whose ESC scores were computed for the age range 46-65 years (Figure 5). Women without MetS had an ESC risk score of 1.2%, whereas those with MetS had an age-standardized risk score of 1.8%. As in men we identified three combinations with lower risk estimates, which are the same as in men, although on an apparently lower risk level. As in men the combinations TG-HDL-BP-GL and WC-TG-HDL-BP resulted in the highest estimated ESC scores (3.0 and 2.1%, resp.), the latter again a combination which was to be found most often within the younger age groups (cf. table 2).

## Discussion

A sufficient sample size of this German-wide cross-sectional study made it possible to calculate all 16 possible combinations of MetS and to analyze these in detail according to sex and an age-group, ranging from 18-99 years. The results of our study show a large variation of the 16 possible combinations with WC-BP-GL as the most frequent combination. An uneven distribution was observed regarding age and sex. Any combination without GL was more frequent in young subjects while any combination with GL was more frequent in the elderly. On the other hand any combination with dyslipidemia (TG, HDL) was more frequent in younger subjects, whereas these combinations were less prevalent in older subjects. The highest impact on an estimated 10-year risk for MI (PROCAM) was observed with TG-HDL-GL-BP in both sexes and TG-HDL-GL in men and TG-HDL-BP in women. However, we could identify combinations with lower or at least equal risks of non-fatal and fatal MI compared to participants without MetS.

### Prevalence of different combination types

The literature concerning combination types in subjects with the MetS is scarce and we identified only few articles dealing with a related topic [11,12,18,28-30]. Thanopoulou and co-workers examined 1,833 randomly selected non-diabetic subjects between 22 and 74 years in five different countries (Algeria, Bulgaria, Egypt, Italy and Greece), measured the prevalence of possible risk factor combinations and compared this to expected frequencies from mathematical modelling [29]. The prevalence of MetS between the countries ranged form 6% to 37%. The most frequent combination type was WC-HDL-BP (31% of all subjects with MetS) followed by TG-HDL-BP (23%) and WC-TG-HDL-BP (19%). Respective frequencies in our sample were 7%, 3%, and 5%. The combination with the highest prevalence in our sample WC-BP-GL with 28% was 0.6% in the Thanopoulou sample. Reasons for these gross differences in prevalence rates might be in part explained by a couple of reasons: (1) our rates for this comparison were not age- and sex-adjusted (GEMCAS: 39% men, age-range 18-99 years) and the data of the Thanopoulou sample were not stratified by sex (50% men), (2) Thanopoulou et al. used a different definition of the MetS, and (3) their sample is considerably younger (ranging from 39 years in Egypt to 50 years in Italy-Pavia) than our GEMCAS sample (52 years). Notably, those combinations that are most frequent in the Thanopoulou sample are those that are most frequent in the younger age-groups in our sample and vice versa. (4) Finally, regarding the unequal distribution of combinations between the sexes and age-groups in our sample, the informative value of the prevalence in Thanopoulou et al. condensed from 6 different study centres with varying sex ratio, age-range and ethnicity, might not be an appropriate comparison in this context here.

Rodriguez-Colon et al. analyzed data from the population based Atherosclerosis Risk in Communities (ARIC) study. About 15,000 stroke-free individuals with 39% having the MetS were followed over 9 years [12]. The prevalence of individual combinations was not reported but the age, gender and race adjusted rates of 7 recombined combination clusters. Combination cluster defined by the presence of either BP or GL or both BP and GL were most prevalent (24%, 23% respectively). Combinations with 4 traits including BP and GL were present in 19% of MetS subjects. The high prevalence of both BP and GL is well compatible with our own data where BP and GL were most frequent at least in the elderly.

Hanefeld and colleagues studied in a representative sample of German patients with type 2 DM the prevalence of the MetS by AHA/NHBLI criteria, its single traits and their combinations [19]. Although a direct comparison to our results is not feasible, it is striking that this group also found a large heterogeneity in the prevalence of the different MetS combinations, with distinct sex differences.

One major difficulty of the comparison of our results with results from these studies is the definition of MetS used. Criteria for defining WC, TG and HDL were identical; the threshold of BP was lower in the studies of Thanopoulou and Rodriguez-Colon (≥135/≥85 vs. ≥130/≥85 mmHg) while the threshold for fasting glucose was ≥110 mg/dl in the Thanopoulou study [29] but ≥100 mg/dl in the Rodriguez-Colon study [12] and ours. Further, Thanopoulou considered antihypertensive drug treatment, Rodriguez-Colon antihypertensive and antidiabetic drug treatment while only antidiabetic drug treatment was considered by us. Finally, individuals with known diabetes were included into Rodriguez-Colon and our study but not in the one by Thanopoulou. In a former analysis we could show that different definitions result in a considerable different prevalence, i.e. the exclusion of pharmacotherapy according to AHA/NHLBI 2004 definition distinctly lowered the prevalence of MetS [20]. Another explanation might be born in the different population investigated, i.e. primary care vs. population based [12,29]. This however would suggest an even higher prevalence of MetS in our study which was not the case (20% in our study, 27% and 39% in those studies). Finally, in our study we performed a complete case analyses and made sure that subjects falling under one combination of the higher order - e.g. four risk factors present - are not simultaneously falling under the combinations with lower order and less criteria - e.g. any three of the above mentioned four. We expect striking differences in the prevalence rate, if this methodological approach is not applied. However, only one publication [29] reported on this methodological aspect.

### Influence of age and gender

Our study demonstrated a substantial influence of age and gender on the prevalence of combinations. Every combination that included GL was more prevalent in the elderly than in young individuals. A particular steep incline in prevalence was observed for combinations that did not include lowered HDL. In an earlier publication of our data we reported that GL was steeply increasing with age while HDL and for men TG showed a strong decline [31]. Thanopoulou [29] and Rodriguez-Colon [12] reported no data on the influence of age or gender on combination prevalence rates beyond documenting a rise of MetS cases with age. Rodriguez-Colon reported however higher rates of elevated WC in women and higher rates of HDL, TG and GL in men. This is identical to our findings, except that low HDL was more frequent in women in our cohort [31].

In our study a particularly steep incline was observed in both men and women with age for WC-BP-GL with slightly lesser inclines for TG-BP-GL and WC-TG-GL (men > women). This is reasonable given that GL gains and HDL looses importance in the elderly [31]. Therefore any combination with GL but not HDL should be highly prevalent in these subjects. On the other hand and this is supported by the data, any combination with low HDL but not GL should be more prevalent in young subjects. This shift in risk factor prevalence could be caused time dependent (years) in that low physical activity may translate into low HDL values first and then lead to insulin resistance in a second step. These assumptions are supported by recent findings from [32].

### MetS combinations and cardiovascular risk

MetS is indicative of an increased risk for cardiovascular disease morbidity and mortality [33]. In Framingham, the MetS alone predicted about 25% of all new-onset cardiovascular disease. In the absence of diabetes, the MetS generally did not rise 10-year risk for CHD to >20%. Ten-year risk in men with MetS generally ranged from 10%-20%. Framingham women with MetS had relatively few CHD events during the course of the 8-year follow-up. The 10-year risk for CHD in most women in this relatively young cohort did not exceed 10%. These data essentially were confirmed in the present dataset with peak risks for MI estimated with the PROCAM algorithm of 15% for men and 4% for women.

In our study all combinations in the younger men were accompanied by a considerably higher risk estimate for MI (PROCAM) compared to men without MetS. Regardless, we found extremely heterogeneously distributed risks for MI. Even more, for fatal MI (ESC) we identified combinations that resulted in distinct lower (although not significant) or equal ESC risk scores and one of these combinations consisted of four traits of MetS (WC-TG-HDL-GL). Our data suggests that the previous assumption - that each combination of the MetS uniformly increases the risk for cardiovascular outcomes - might not hold true and supports the findings of a French [28] and the Framingham offspring study [30]. Therefore, it is not surprising that controversy exists over whether a diagnosis of MetS provides more useful information about CVD risk than its individual components do. An alternative has been proposed by Franks and Olsson [9], suggesting to include only the features that are uniquely informative and to weight each of these components by an empirical value. Of course this supposition must be examined in prospective studies, since our cross-sectional approach might have introduced bias in terms of the temporal occurrence of a parameter value (i.e. waist circumference) and CVD.

There was however a strong variation of the estimated risk in relation of the combination detected in a particular individual. Low HDL was a frequent component of combinations in high risk subjects of either gender for MI (PROCAM) and cardiovascular death (ESC score). GL was highly prevalent in men but not so in women. Elevated blood pressure was a component in many high risk combination types. Rodriguez-Colon et al. [12] analyzed the impact of combinations on the incidence of stroke in about 15,000 subjects and found 1) a linear relationship between the number of risk factors and the risk of incident stroke and 2) that persons with combination types including either elevated BP or GL had the highest risk for incident stroke (hazard ratio 2.7-4.2) than MetS without these 2 components (hazard ratio ≤2.0). Although we also included individuals with stroke (3.7% of men and 2.0% women with stroke/TIA in the past [34]) and investigated a primary care sample our results fit with these observations and enhance these by data on the incidence of myocardial infarction and cardiovascular disease related death.

### Strength and Limitation

The strengths of the study are the sample size, the nationwide approach and the assessment of the MetS as primary study target, so comprising all required variables as original measures. However, the participants are strictly speaking not a real population-based sample, but close to being so. Some participants meeting MetS criteria might avoid attending their physician because they are in denial, in this case an underestimation of the prevalence would have been occurred. On the other hand, the estimated prevalence might be too high because the healthy population does not routinely visit their physician. However, 91.8% of the adult persons in Germany consult a general practitioner during one year [35]. Furthermore, characteristics of our sample are comparable to other German population-based samples and to German federal statistical data i.e. with regard to anthropometric measures, smoking status, marital status, schooling, and unemployment rate, the latter which was in Germany in October 2005 10.4%, in our study based on self-reports 10.2% after adjusting for structural differences. However, the proportion of participants with chronic diseases (diabetes, CVD) is higher than compared to population-based sample, but still lower than real patient-based samples [36].

A further possible limitation of our approach to base risk estimates on PROCAM [26] and ESC scores [27] deserves notion. The definition of the MetS is of particular diagnostic value if it indicates an incremental risk over adding the unique traits of the MetS into a usual risk scoring model. This cannot be shown by our cross-sectional design. For the article on the definition of the MetS by Grundy et al. [4] it was tested whether the MetS carries an incremental risk beyond the usual risk factors of the Framingham algorithm. They found that most of the MetS associated risk was well captured by age, blood pressure, total cholesterol, diabetes and HDL without any significant further contribution of other risk factors.

## Conclusions

The data of GEMCAS provide an ample sample to explore the combination types of the MetS. Although about on third of the study population was diagnosed as having the MetS based mainly on increased WC, elevated BP, and impaired fasting GL, there was still a large variation in the prevalence of the possible 16 combination types and their association to cardiovascular risk. The importance of different combinations of MetS changes with age and between genders putting emphasis on a tailored approach towards very young or very old subjects. This knowledge may guide clinicians to effectively screen individuals and prioritize diagnostic procedures depending on age and gender.

## Competing interests

The authors declare that they have no competing interests.

## Authors' contributions

SM planned and performed the study and wrote the manuscript. CB, CL and LG performed the statistical analysis. BvS and JW participated in the study design; PB has been revising the manuscript critically for important intellectual content. KHJ supervised scientific, ethical and data privacy issues of the study. All authors read and approved the final manuscript.

## Authors' information

BvS is employee of Sanofi Aventis Deutschland GmbH.
